# Hyperbaric Oxygen Therapy Improved Neovascularisation Following Limb Ischaemia—The Role of ROS Mitigation

**DOI:** 10.1111/jcmm.70310

**Published:** 2024-12-25

**Authors:** You‐Cheng Lin, Jhih‐Yuan Shih, Yu‐Wen Lin, Ko‐Chi Niu, Chon‐Seng Hong, Zhih‐Cherng Chen, Shin‐Chen Pan, Tzu‐Yen Chang, Wei‐Chih Kan, Wei‐Ting Chang

**Affiliations:** ^1^ Division of Plastic Surgery, Department of Surgery Chi‐Mei Medical Center Tainan Taiwan; ^2^ Division of Cardiology, Department of Internal Medicine Chi Mei Medical Center Tainan Taiwan; ^3^ Department of Cardiology Chi Mei Medical Center Tainan Taiwan; ^4^ School of Medicine, College of Medicine National Sun Yat‐sen University Kaohsiung Taiwan; ^5^ Department of Hyperbaric Oxygen Medicine Chi Mei Medical Center Tainan Taiwan; ^6^ Department of Health and Nutrition Chia Nan University of Pharmacy and Science Tainan Taiwan; ^7^ Department of Surgery, Section of Plastic and Reconstructive Surgery, College of Medicine, National Cheng Kung University Hospital National Cheng Kung University Tainan Taiwan; ^8^ Division of Nephrology, Department of Internal Medicine Chi Mei Medical Center Tainan Taiwan; ^9^ Department of Medical Laboratory Science and Biotechnology Chung Hwa University of Medical Technology Tainan Taiwan; ^10^ Department of Radiology Chi‐Mei Medical Center Tainan Taiwan; ^11^ School of Medicine and Doctoral Program of Clinical and Experimental Medicine, College of Medicine and Center of Excellence for Metabolic Associated Fatty Liver Disease National Sun Yat‐sen University Kaohsiung Taiwan

**Keywords:** angiogenesis, HBO, limb ischaemia, ROS

## Abstract

Hyperbaric oxygen (HBO) therapy has emerged as a potential treatment, shown to enhance blood flow and angiogenesis. However, specific effects and mechanisms of HBO on limb ischaemia responding to a hypoxic environment remain largely unknown. We aimed to investigate the therapeutic potential of HBO in the treatment of limb ischaemia. Following limb ischaemia surgery, we evaluated the angiogenic capacity in wild‐type C57BL/6J mice subjected to HBO treatment (100% oxygen at 3 ATA for 1 h/day for five consecutive days) compared to untreated controls. Notably, through laser Doppler perfusion imaging and CD31 staining mice receiving HBO postlimb ischaemia surgery exhibited significantly enhanced angiogenic capability and reduced ROS expression compared to nontreated counterparts. Additionally, in vitro experiments were conducted to investigate whether HBO could mitigate endothelial cell dysfunction and reactive oxygen species (ROS) production triggered by oxygen–glucose deprivation (OGD). HBO treatment rescued the impaired proliferation, migration and tube formation of endothelial cells following OGD. Mechanistically, HBO upregulated the expression of proangiogenic proteins, including vascular endothelial growth factor (VEGF), haem oxygenase‐1 (HO‐1), hypoxia‐inducible factor 1 (HIF‐1) and nuclear factor erythroid 2–related factor 2 (Nrf2). Collectively, HBO treatment shows promise in augmenting the endogenous angiogenic potential and suppressing ROS levels in limb ischaemia.

## 1 Introduction

With its escalating prevalence, peripheral artery disease (PAD) has garnered significant attention [1]. Despite advancements in surgical and endovascular interventions for PAD treatment, the longevity of limb vessel patency remains restricted [2]. The prognosis of PAD‐induced limb ischaemia is determined by not only atherosclerosis but anomalous vascular endothelial cell proliferation and angiogenesis [3, 4]. Angiogenesis, the process of blood vessel growth and proliferation from existing vasculature, augments the microvascular density of occluded arteries, thus improving perfusion [4]. To note, limb ischaemia–induced hypoxia could trigger the production of reactive oxygen species (ROS) given to an imbalance in cellular redox status. Excessive ROS production in ischaemic tissues can lead to oxidative stress, causing damage to endothelial cells lining blood vessels [5, 6]. ROS may inhibit the activity of vascular endothelial growth factor (VEGF), a key regulator of angiogenesis, by disrupting downstream signalling pathways or by directly modifying VEGF receptors on endothelial cells. Beyond VEGF, haem oxygenase‐1 (HO‐1), hypoxia‐inducible factor‐1 (HIF‐1), kelch‐like ECH–associated protein 1 (Keap1) and nuclear factor erythroid 2–related factor 2 (Nrf2) pathway also play crucial roles in the cellular response to oxidative stress [7, 8, 9]. This inhibition of proangiogenic signalling pathways hampers the initiation and progression of angiogenesis in ischaemic tissues. Understanding the mechanisms by which ROS suppress angiogenesis is important for developing therapeutic strategies aimed at promoting revascularisation and improving outcomes in patients with peripheral artery disease and limb ischaemia.

Hyperbaric oxygen (HBO) therapy involves the administration of pure oxygen under elevated atmospheric pressure, typically ranging from 2.5 to 3 atmospheres absolute (ATA) for a duration of 60–90 min. This pressurised environment is created within a chamber using either pressurised air or oxygen. In limb ischaemic settings, HBO has found application in treating patients with limb ischaemia. This is attributed to its capacity to augment the oxygen levels in hypoperfused tissues. The heightened oxygen content in hypoxic tissues, in turn, facilitates and enhances the process of ischaemic repair [10]. Nevertheless, the effect of HBO on endothelial cells in response to a hypoxic environment is largely unknown. Herein, we aimed to investigate the regulatory mechanism and therapeutic potential of HBO in the treatment of limb ischaemia.

## 2 Materials and Methods

### 2.1 Animal Model of Limb Ischaemia

All animal trials were authorised and executed in compliance with the rigorous regulations set by the Subcommittee on Research Animal Care at Chi‐Mei Medical Center. The procedures followed the guidelines outlined in the ‘Guide for the Care and Use of Laboratory Animals’ and have been approved by the animal ethical committee (Ethical code IACUC 112‐30). Also, to achieve adequate sample number for each experimental condition, a block randomisation method was used to assign experimental animals to groups. Adult male C57BL/6J mice, aged 12 weeks, were randomly assigned to one of three groups: (1) Sham group (*n* = 8 biologically independent animals), (2) limb ischaemia group (*n* = 10 biologically independent animals) and (3) HBO + limb ischaemia group (n = 10 biologically independent animals). Limb ischaemia was induced in the left limb of mice as previously described [11, 12]. Briefly, C57BL/6J mice underwent anaesthesia via intraperitoneal injection of pentobarbital (80 mg/kg). Following anaesthesia, left femoral artery ligation and excision were performed. Sham surgery involved exposure of the femoral artery and vein without ligation or excision. In the HBO + limb ischaemia group, the mice were placed in an HBO chamber and exposed to 100% oxygen at 3 ATA for 1 h/day for five consecutive days on day 1 after surgery.

### 2.2 Laser Doppler Perfusion Imaging

Limb blood flow in mice was evaluated using a sequential laser Doppler imaging system (PeriScan PIM 3 Systems; Perimed AB, Sweden) on days 0, 1, 7, 14, 21 and 28. The laser Doppler perfusion imaging index was calculated as the ratio of perfusion in the ischaemic limb to that in the nonischaemic limb. After the end of the experiment, calf muscles were harvested, and the reduction in mass of the ischaemic muscle was quantified by expressing it as the ratio of ischaemic (left) calf muscle to nonischaemic (right) calf muscle.

### 2.3 Capillary Density in Ischaemic Limb Analyses

Capillary density within ischaemic (left) calf muscles was quantified using immunofluorescence analysis. At 28 days postlimb ischaemia, muscle samples were embedded in paraffin and processed as previously described [13, 14]. Tissue sections from the left calf of each mouse were stained with primary antibodies against CD31 (1:100; Abcam, Cambridge, MA, USA) and secondary antibodies conjugated to Alexa594 (1:500; Abcam, Cambridge, UK) in the dark. Nuclei were stained with 4′,6‐diamidino‐2‐phenylindole (DAPI; Abcam, Cambridge, MA, USA). Images were captured using a fluorescence microscope (Olympus BX51; Olympus Optical Co. Ltd., Tokyo, Japan). Quantitative assessment of capillary density involved counting the average number of capillaries expressing positive CD31 on endothelial cells.

### 2.4 Immunofluorescence Staining of 8‐Hydroxy‐29‐Deoxyguanosine (8‐OHdG), VEGF and HIF‐1α

Oxidative stress in endothelial cells was assessed by quantifying the muscle immunoreactivity to 8‐OhdG. The tissue sections were incubated with antibodies to CD31 (1:100; Abcam, Cambridge, MA, USA) and 8‐OhdG monoclonal antibody (Bioss Antibodies, Woburn, MA, USA) following the manufacturer's protocol. Also, VEGF (1:100; Merck Millipore, Billerica, MA, USA) or HIF‐1α (1:100; Abcam, Cambridge, MA, USA) were costained with CD31 with the secondary antibodies conjugated to Alexa488 (1:400; Abcam, Cambridge, UK) and Alexa594 (1:500; Abcam, Cambridge, UK) respectively. Nuclei were counterstained with DAPI. Images were captured using a fluorescence microscope (Olympus BX51; Olympus Optical Co. Ltd., Tokyo, Japan). The percentage of ROS in endothelial cells was quantified, and the results are presented as the ratio of positive 8‐OhdG to CD31^+^ cells.

### 2.5 Cell Culture

Human umbilical vein endothelial cells (HUVECs) were obtained from ATCC (University Boulevard Manassas VA, USA). The cells were cultivated in standard endothelial cell growth medium (M200; Gibco, Thermo Fisher Scientific, Massachusetts, USA) supplemented with 10% foetal bovine serum, 100 units/mL penicillin and 50 units/mL streptomycin (Invitrogen, Thermo Fisher Scientific, Massachusetts, USA) in a 37°C, 5% CO_2_ incubator. For the ischaemic condition simulation in the limb ischaemia model, HUVECs were exposed to oxygen–glucose deprivation (OGD; 95% N_2_/5% CO_2_, no glucose, no serum) for 3 h [15]. Following OGD injury, the HUVECs were treated with HBO (100% oxygen at 3 ATA) for 1 h.

### 2.6 Bromodeoxyuridine (BrdU) Cell Proliferation Assay

The proliferation of HUVEC was evaluated using the BrdU Cell Proliferation Assay Kit (Abcam, Cambridge, MA, USA) in 96‐well plates, following the manufacturer's recommended protocol. Initially, 2 × 10^5^ HUVEC cells were seeded into the wells and allowed to grow for 24 h at 37 °C. Subsequently, cells were treated with OGD for 3 h followed by HBO (100% oxygen at 3 ATA) for 1 h. BrdU was added to the medium, and incubation continued for an additional 24 h under either normoxia or hypoxia conditions. After the incubation period, cell proliferation was determined using a spectrophotometer (MULTISKAN. GO, Thermo Fisher Scientific, CA, USA) at a wavelength of 450 nm.

### 2.7 Migration Assay

HUVEC were seeded in a culture‐insert (ibidi culture‐insert 2 well, ibidi GmbH, Martinsried, Germany) at a density of 2 × 10^5^. Following a 24‐h incubation, the cells were exposed to OGD for 3 h followed by HBO (100% oxygen at 3 ATA) for 1 h. Then, the culture‐insert was removed, washed with PBS and recultured with culture medium containing 10% FBS. The rate of cell migration was monitored for 6 h by capturing images with a light microscope (Olympus BX51; Olympus Optical Co. Ltd., Tokyo, Japan) at 0 and 6 h. Quantitative analysis of cell migration distance was carried out using Image J software. At least three independent experiments were performed.

### 2.8 Tube Formation Assay

The angiogenic activity of HUVECs was measured by Matrigel tube formation assay. Cells were treated with OGD for 3 h and then subcultured to 24‐well plates with Matrigel (50 μL/well, CORNING, Thermo Fisher Scientific, CA, USA) followed by HBO (100% oxygen at 3 ATA) for 1 h. Subsequently, the HUVEC were seeded in Matrigel basement membrane matrix and images of tube formation were captured after 16 h under an inverted microscope at 100× magnification (Olympus BX51, Olympus Optical Co. Ltd., Tokyo, Japan). The tube number of branches was measured by ImageJ software (National Institutes of Health, Bethesda, MD, USA).

### 2.9 ROS Measurement

ROS generation in HUVEC was assessed using the fluorescent dihydroethidium probe (DHE, Abcam, Cambridge, MA, USA) according to the manufacturer's protocol. Briefly, cells cultured on glass slides in plates underwent various conditions: (1) Control group; (2) OGD group; (3) OGD + HBO group. After treatment, cells were washed with PBS and then incubated with DHE in DMEM for 30 min at 37°C. Subsequently, the cells were washed with PBS, and fluorescence images were captured using a fluorescence microscope (Olympus BX51; Olympus Optical Co. Ltd., Tokyo, Japan).

### 2.10 Western Blotting

Calf muscle tissue or HUVEC were homogenised in lysis buffer to extract proteins. Total protein concentration was measured using the BCA protein assay (Thermo Fisher Scientific, CA, USA). Equal amounts of protein were subjected to 8%–15% sodium dodecyl sulphate–polyacrylamide gel electrophoresis (SDS‐PAGE) and transferred to polyvinylidene fluoride (PVDF) membranes (MERCK Millipore, Massachusetts, USA). Nonspecific protein binding was prevented by blocking with 5% milk in Tris‐buffered solution (pH 7.6) at room temperature for 1 h. After blocking, the membranes were incubated with primary antibodies, including VEGF (1:1000; Merck Millipore, Billerica, MA, USA), VEGFR2 (1:1000; Abcam, Cambridge, MA, USA), Erk (1:1000; Cell Signalling, Massachusetts, USA), Nrf2 (1:1000; GeneTex, Irvine, CA, USA), Keap1 (1:1000; Cell Signalling, Massachusetts, USA), HO‐1 (1:1000; Cell Signalling, Massachusetts, USA), HIF‐1α (1:1000; Abcam, Cambridge, MA, USA) or glyceraldehyde‐3‐phosphate dehydrogenase (GAPDH) (1:5000; Sigma‐Aldrich Co., St Louis, MO, USA) overnight at 4°C. Following primary antibody incubation, membranes were treated with a horseradish peroxidase–conjugated secondary antibody (1:5000; MERCK Millipore, Massachusetts, USA, and Sigma‐Aldrich Co., St Louis, MO, USA) for 1 h at room temperature. Immunoreactive protein bands were detected using the ECL‐Western blotting system (AVEGENE CHEMX 400). Protein expression levels were normalised to GAPDH. In brief, we measure the band intensities of the target protein and GAPDH using software like ImageJ (Bethesda, NIH, USA). After subtracting the background intensity from each band, then we calculate the ratio of the target protein intensity to GAPDH intensity for each sample. As described previously [16], this approach provides a reliable method to quantify protein levels normalised to a housekeeping control like GAPDH.

### 2.11 RNA Isolation and Quantitative Reverse Real‐Time Polymerase Chain Reaction (RT‐PCR)

Total RNA was isolated using Trizol reagent (Ambion) following the manufacturer's instructions. cDNA synthesis was performed with TaqMan MicroRNA Assays (Foster City, CA). The primer sequences used for RT‐PCR targeting VEGF, HIF1A and β‐actin were as follows: *VEGF*: Forward: ctggagcgtgtacgttggtg, Reverse: acacgtctgcggatcttgta; *HIF1A*: Forward: actcatccatgtgaccacg, Reverse: tagttctcccccggctag; *β‐actin*: Forward: cctgactgactacctcatgaag, Reverse: gacgtagcacagcttctcctta (Mission Biotech, Taipei, Taiwan). mRNA levels of abovementioned genes were quantified using the 7500 Fast Real‐Time PCR System (Applied Biosystems, Foster City, CA, USA). As previously described, intracellular microRNA expression levels were normalised to GAPDH. Final fold expression changes were calculated using the 2^−ΔΔCt^ method.

### 2.12 Statistical Analysis

The data are presented as the mean ± standard errors for three or more independent experiments. All the data were analysed using GraphPad Prism 6.01 software (La Jolla, CA, USA). Statistical differences among groups were determined using one‐way ANOVA with Tukey's multiple comparisons test. A value of *p* < 0.05 was considered to be statistically significant.

## 3 Results

### 3.1 HBO Therapy Improves Perfusion Recovery in the Limb Ischaemia Model

**FIGURE 1:**
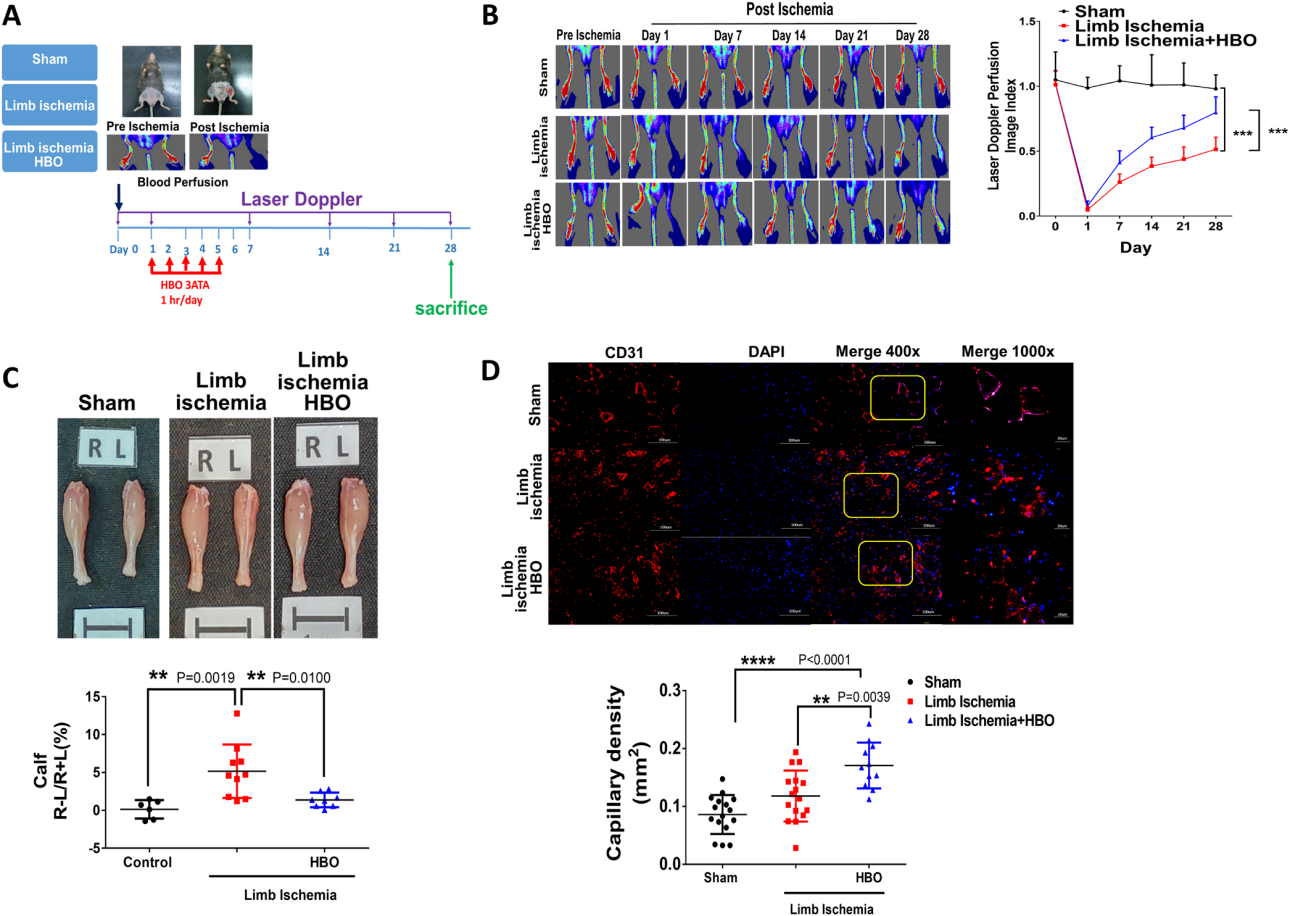
Hyperbaric Oxygen (HBO) enhances angiogenesis in mice postlimb ischaemia. (A) The study design illustrating limb ischaemia mice with or without HBO treatment including three groups: (1) Sham group (*n* = 8 biologically independent animals), (2) limb ischaemia group (*n* = 10 biologically independent animals) and (3) HBO + limb ischaemia group (*n* = 10 biologically independent animals). In the HBO + limb ischaemia group, mice were placed in a HBO chamber and exposed to 100% oxygen at 3 ATA for 1 h per day over five consecutive days, beginning on the first day postsurgery. (B) Representative images and quantification of laser Doppler perfusion flow in limb ischaemia mice treated with or without HBO treatment. (C) Representative images and weight quantifications of harvested ischaemic (L) limbs compared with nonischaemic (R) limbs. (D) Representative images and quantification of CD31 immunostaining representing capillary density. Data are presented as mean ± SEM. One‐way ANOVA followed by Tukey's test was used to compare groups. **p < 0.01, ***p < 0.005 and ****p < 0.001 compared with the indicated groups.

We established a murine limb ischaemia model to explore the effect of HBO therapy on functional recovery and angiogenesis following ischaemic events. Although no significant changes in body weight and food intake were observed among sham mice and limb ischaemia mice treated with or without HBO, the water intake increased in those postlimb ischaemic surgeries (Figure S1). After the surgery of limb ischaemia, mice underwent HBO therapy at 3 ATA for 1 h per day over five consecutive days and using LDPI we monitored the limb blood flow for up to 28 days (Figure 1A). On day 28, limb perfusion showed a significant recovery in mice subjected to HBO therapy compared to those without HBO postlimb ischaemia (Figure 1B). Likewise, calf weight loss was attenuated in mice receiving HBO (Figure 1C). Further, immunofluorescence staining of CD31 in mouse calf muscle sections revealed that although limb ischaemia injury partially augmented the capillary density, HBO therapy even more significantly increased the CD31 expressions in mice compared with those without HBO therapy (Figure 1D).

### 3.2 HBO Therapy Inhibits ROS and Increases Angiogenesis Associated With the Erk/Nrf2/HO‐1 Pathway in a Mouse Model of Limb Ischaemia

**FIGURE 2:**
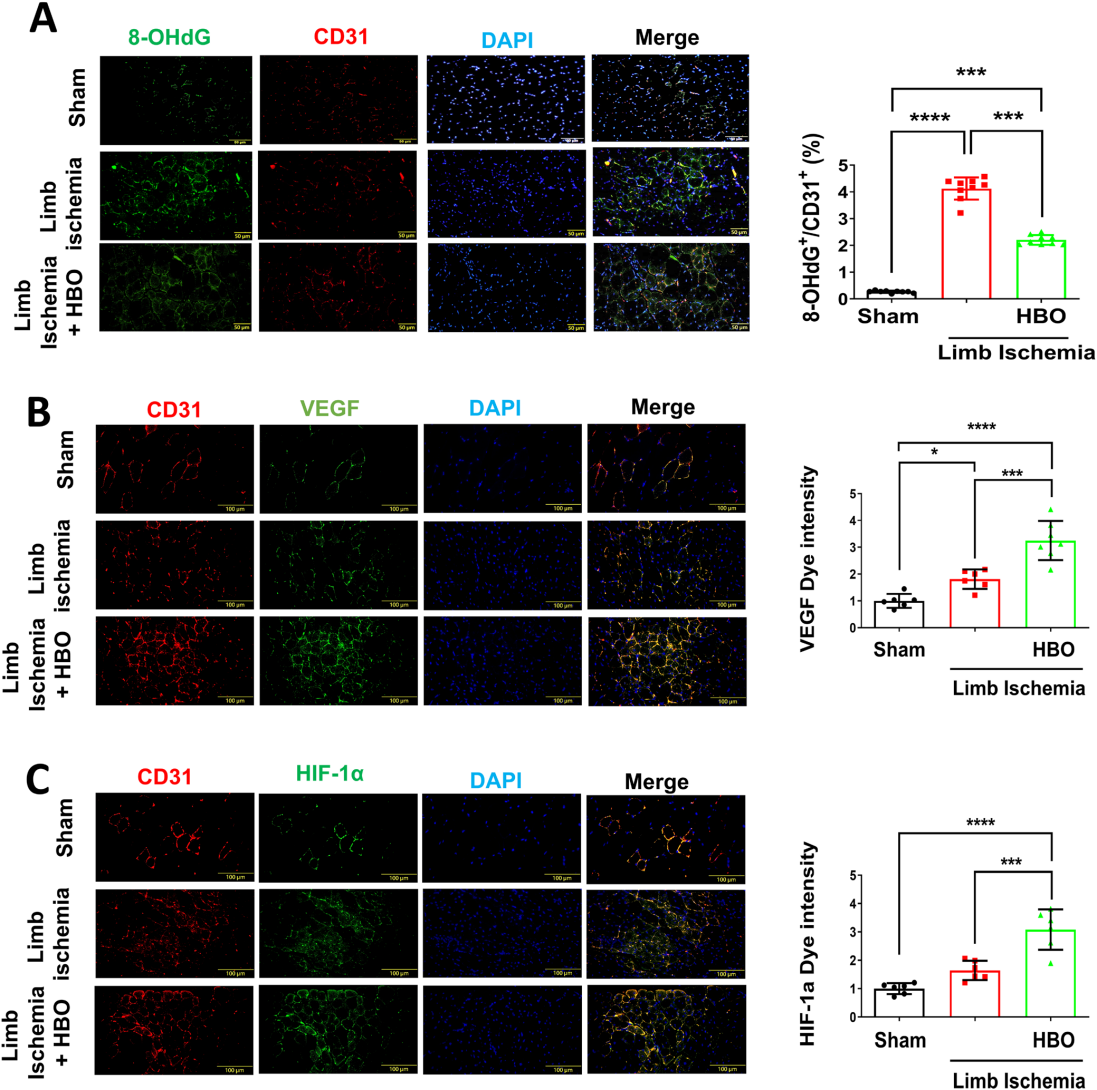
Hyperbaric Oxygen (HBO) alleviates ROS accumulation and boosts HIF‐1α and VEGF activities in mice with limb ischaemia. Double immunostaining with endothelial marker CD31, ROS marker 8‐OHdG, HIF‐1α and VEGF in the left calf muscle mice following limb ischaemia surgery, with or without HBO therapy. Representative images and quantification of (A) 8‐OHdG (B) VEGF and (C) HIF‐1α expressions in endothelial cells (red; CD 31 positive). Cell nuclei were stained with DAPI (blue). Scale bar: 50 μm. Data are presented as mean ± SEM. One‐way ANOVA followed by Tukey's test was used to compare groups. *p < 0.05, *** *p* < 0.005 and ****p < 0.001 compared with the indicated groups.

**FIGURE 3:**
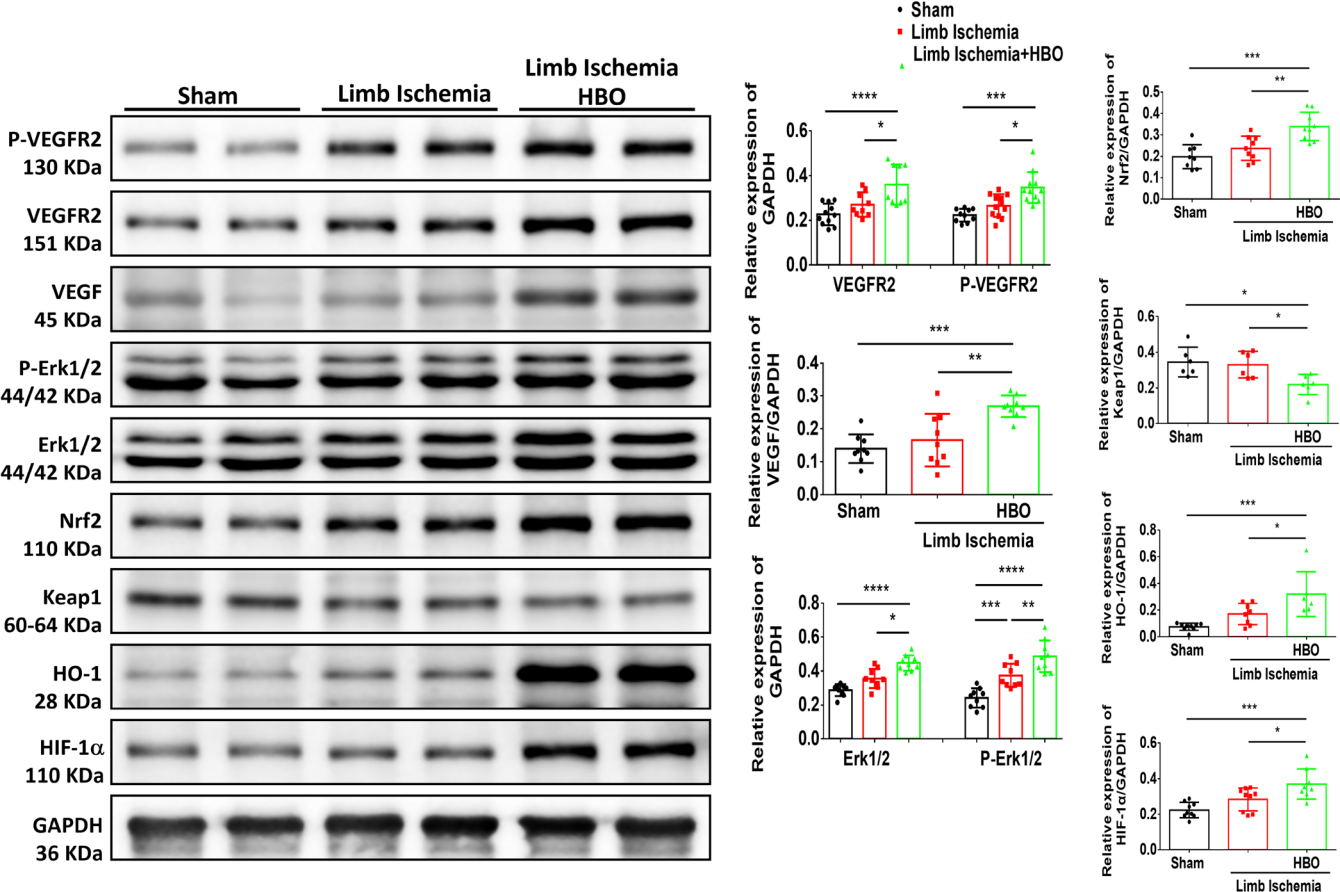
Hyperbaric Oxygen (HBO) augments angiogenesis‐associated protein in mice with limb ischaemia surgery. Representative blots and quantification of VEGFR, VEGF, Erk, Nrf2, HO‐1 and HIF‐1α in the left limbs of mice treated with or without HBO therapy. Data are presented as mean ± SEM. One‐way ANOVA followed by Tukey's test was used to compare groups. *p < 0.05, **p < 0.01, ***p < 0.005 and ****p < 0.001 compared with the indicated groups.

Previous literature suggests that dysregulation of ROS levels in ischaemic tissues can disturb the intricate balance between pro‐ and antiangiogenic signals, ultimately suppressing angiogenesis in limb ischaemia [6, 17]. Therefore, we assessed ROS expression in the ischaemic calf muscle of each group using 8‐OHdG immunohistological staining. Compared to the sham group, mice subjected to limb ischaemia injury exhibited elevated ROS levels in CD31‐positive endothelial cells, which were mitigated by HBO therapy (Figure 2A). Also, immunostaining for VEGF, a key regulator of angiogenesis (Figure 2B), and HIF‐1α, a crucial regulator in oxidative stress (Figure 2C), showed significant upregulations in HUVECs post‐HBO treatment. Additionally, we investigated the molecular mechanism of HBO in angiogenesis by measuring the expression levels of angiogenesis‐associated proteins in limb muscles using Western blot analysis. As shown in Figure 3, we compared the expressions of angiogenesis and oxidative stress–associated proteins including VEGFR, VEGF, Erk, Nrf2, Keap1, HO‐1 and HIF‐1α in the left limbs of the sham mice, mice postlimb ischaemic surgeries and those postlimb ischaemic surgeries receiving HBO therapies. Notably, the expression of angiogenesis‐associated proteins in the ischaemic calf muscle of mice undergoing HBO therapy was higher compared to mice without HBO therapy. Taken together, our findings imply the beneficial effect of HBO therapy in reducing ROS expression induced by limb ischaemia and enhancing angiogenesis associated with the Erk/Nrf2/HO‐1 pathway.

### 3.3 HBO Improves Proliferation, Migration and Tube Formation in HUVECs Under OGD Injury

**FIGURE 4:**
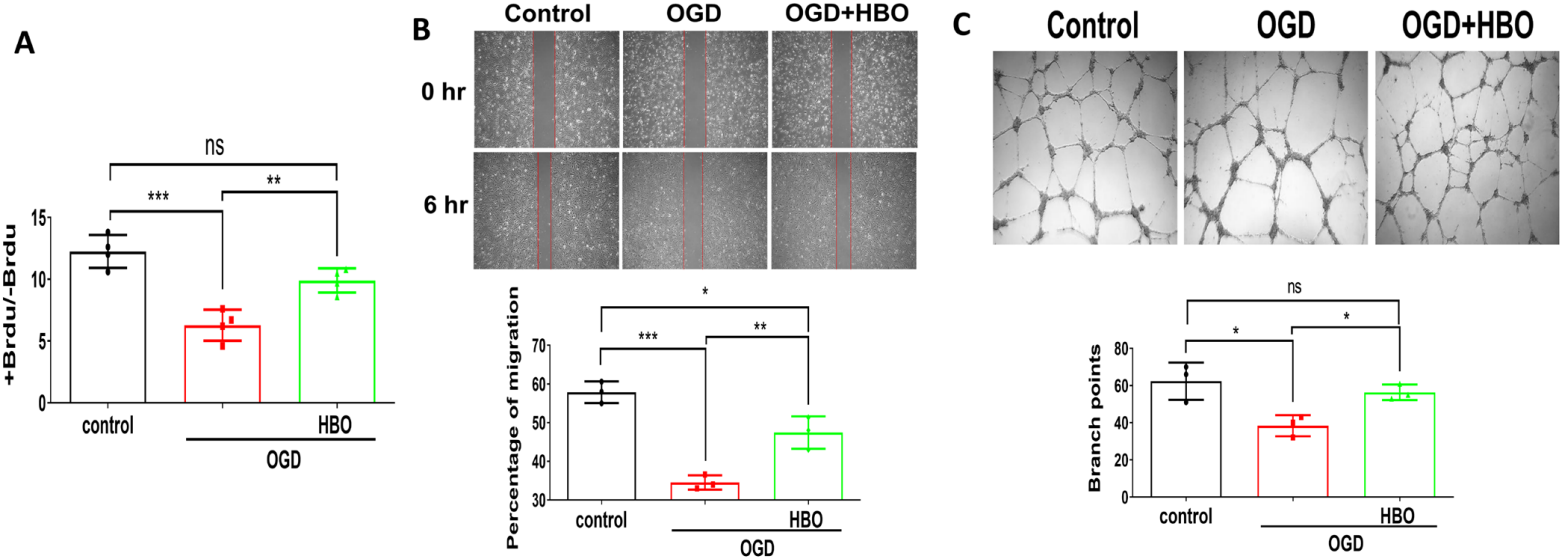
Hyperbaric Oxygen (HBO) enhances proliferation, migration and tube formation in human umbilical vein endothelial cells (HUVECs) under oxygen–glucose deprivation (OGD) injury. Following OGD injury (95% N_2_/5% CO_2_, no glucose, no serum) for 3 h, the HUVECs were treated with HBO (100% oxygen at 3 ATA) for 1 h. (A) Cell proliferation evaluated by measuring BrdU incorporation into cells for 24 h, (B) cell migration for 6 h and (C) tube formation for 16 h evaluated by the branched points in HUVECs. The experiment was conducted in triplicate. Data are presented as mean ± SEM. One‐way ANOVA followed by Tukey's test was used to compare groups. *p < 0.05, **p < 0.01 and ***p < 0.001 compared with the indicated groups.

We established a cellular model mimicking limb ischaemia using HUVEC exposed to OGD conditions. Under OGD conditions, a notable reduction in cell proliferation, migration and tube formation in HUVEC. Conversely, HBO therapy exhibited a marked improvement in cell proliferation, migration and tube formation in HUVECs (Figure 4).

### 3.4 HBO Inhibits ROS and Enhances Angiogenesis in HUVECs Under OGD Injury

**FIGURE 5:**
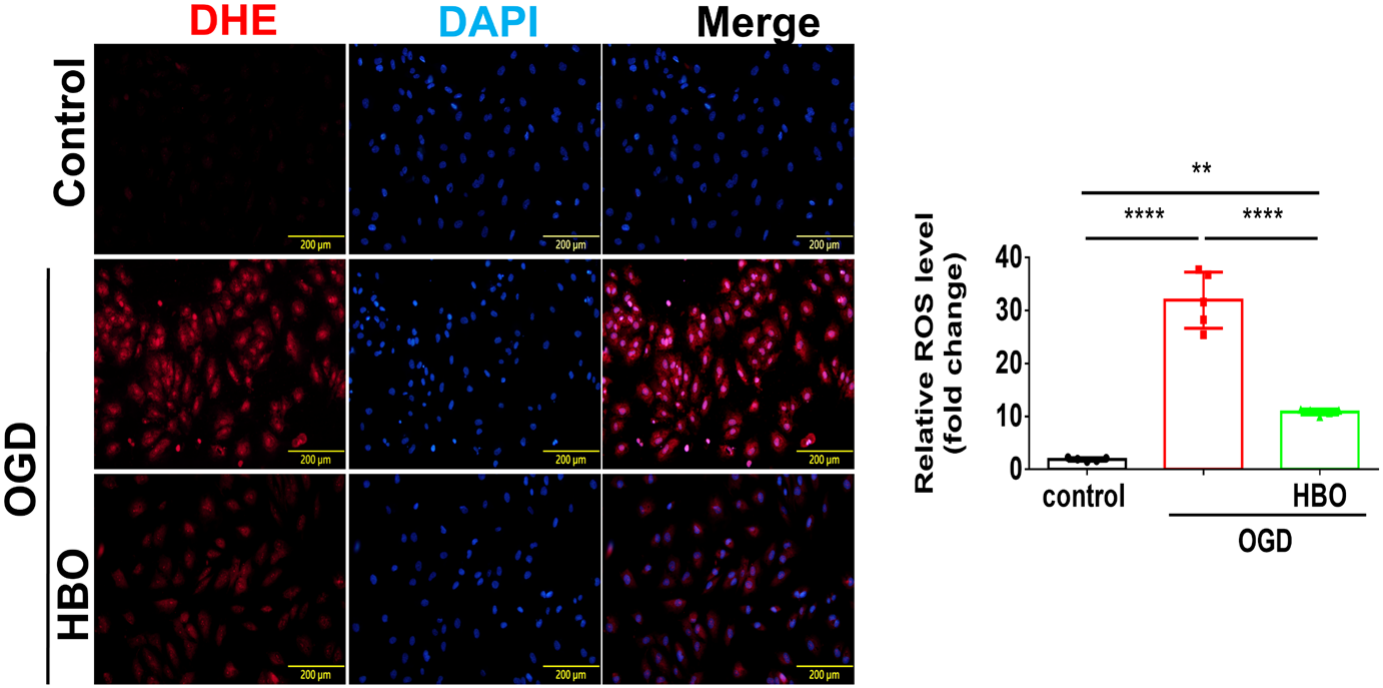
Hyperbaric Oxygen (HBO) attenuates reactive oxygen species (ROS) generation in the human umbilical vein endothelial cells (HUVECs) under oxygen–glucose deprivation (OGD) injury. After HUVECs were subjected to OGD injury for 3 h, HUVECs were treated with or without HBO therapy (100% oxygen at 3 ATA) for 1 h. The intracellular expression of ROS was measured by dihydroethidium (DHE) immunohistological staining. The experiment was conducted in triplicate. Data are presented as mean ± SEM. One‐way ANOVA followed by Tukey's test was used to compare groups. **p < 0.0001 and ****p < 0.0001 compared with the indicated groups.

**FIGURE 6:**
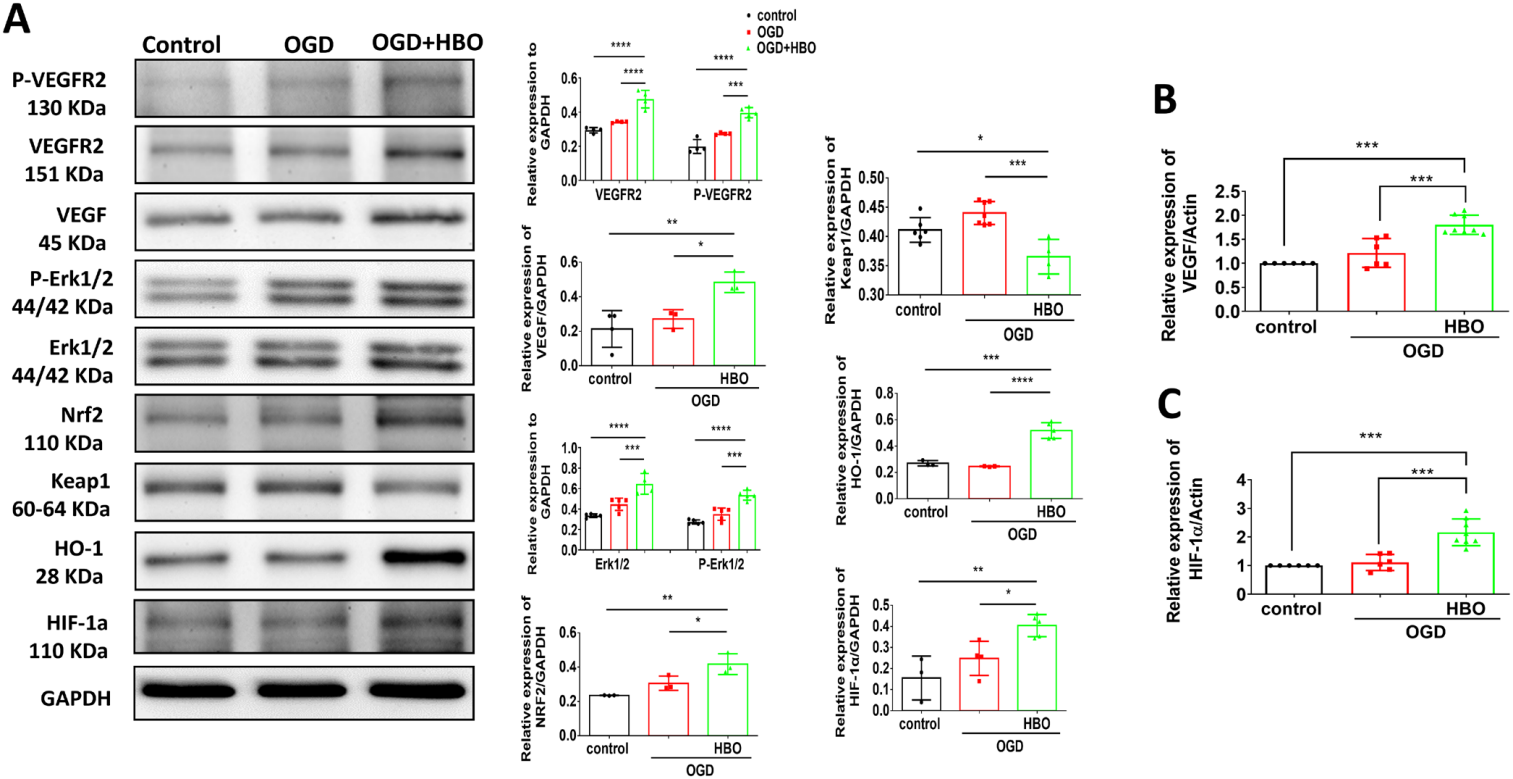
Hyperbaric Oxygen (HBO) improves angiogenesis‐associated mRNA and protein expressions in human umbilical vein endothelial cells (HUVECs) under oxygen–glucose deprivation (OGD) injury. Under OGD conditions for 3 h, HUVECs were treated with or without HBO (100% oxygen at 3 ATA) for 1 h. (A) Representative blots and quantification of VEGFR, VEGF, Erk, Nrf2, HO‐1 and HIF‐1α in HUVECs treated with or without HBO therapy. Transcriptions of (B) VEGF and (C) HIF‐1α detected by reverse transcription‐PCR (RT‐PCR). The experiment was repeated in triplicate. Data are presented as mean ± SEM. One‐way ANOVA followed by Tukey's test was used to compare groups. *p < 0.05, **p < 0.01, ***p < 0.001 and ****p < 0.0001 compared with the indicated groups.

The level of ROS in HUVEC was measured using DHE staining, revealing that HBO therapy mitigated the OGD‐induced increase in intracellular ROS expression (Figure 5). Additionally, angiogenesis‐associated protein was detected by Western blotting. OGD‐treated HUVECs exhibited an upregulation in the expression of angiogenesis‐related proteins, including VEGFR, VEGF, Erk, Keap1, Nrf2, HO‐1 and HIF‐1α. The levels of angiogenesis‐related proteins in HUVECs subjected to HBO treatment were significantly higher than those of HUVECs without HBO treatment (Figure 6A). Also, transcriptions of angiogenesis‐related mRNA including VEGF and HIF‐1α significantly increased in HUVECs subjected to HBO (Figure 6B,C). Taken together, in our cell model of OGD‐induced endothelial cell injury, treatment with HBO significantly decreased OGD‐triggered ROS and improved angiogenesis.

## 4 Discussion

**FIGURE 7:**
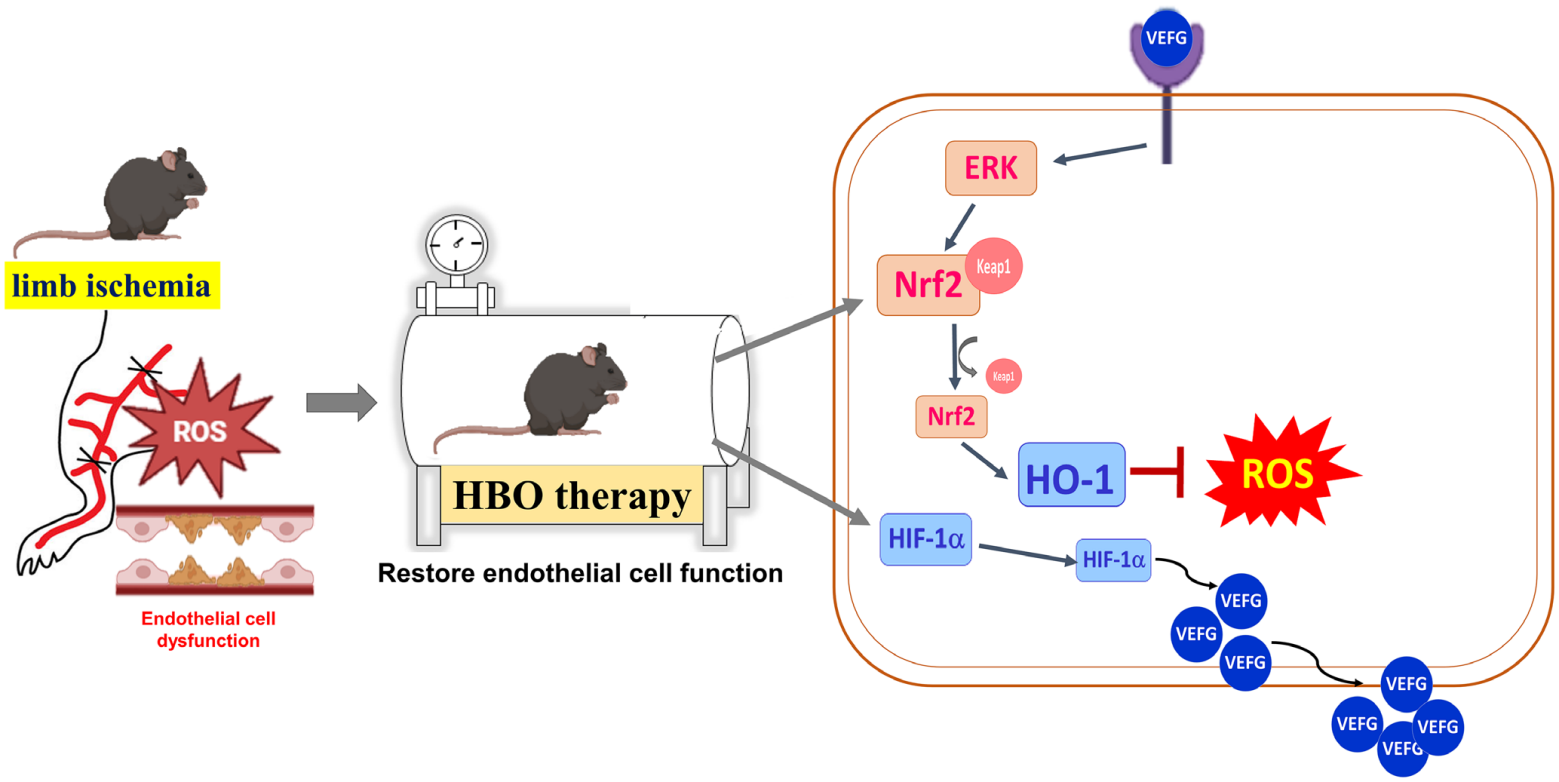
Summary of HBO preventing limb ischemia‐induced endothelial cell dysfunction.

This study highlights the therapeutic benefits of HBO therapy in improving blood flow and functional recovery in cases of limb ischaemia. By clarifying the significance of reducing ROS and activating the HO‐1/Nrf2/Keap1 pathway, we offer valuable insights into the underlying mechanisms of HBO therapy. These findings demonstrate that HBO treatment could alleviate limb ischaemia and is summarised in Figure 7.

Limb ischaemia–induced hypoxia can trigger the production of ROS in the affected area [18, 19]. While low levels of ROS often positively stimulate angiogenic events, excessive ROS can be detrimental [20]. They contribute to endothelial dysfunction, impair neovascularisation and induce apoptosis in endothelial cells participating in angiogenesis, thereby depleting the requisite cell population for new blood vessel formation and suppressing angiogenesis in ischaemic tissues [5, 21]. ROS‐induced inhibitory molecules, such as hydrogen peroxide and superoxide anions, can also inhibit endothelial cell function and disrupt angiogenic signalling pathways, contributing to the suppression of angiogenesis in limb ischaemia [17, 22]. Excessive accumulation of hydrogen peroxide, which induces endothelial cell apoptosis and inhibits angiogenesis, while also directly impeding endothelial cell migration and tube formation, further disrupts the angiogenic process [23]. Loo et al. by using a mouse wound model found that a high concentration of H_2_O_2_ delayed angiogenesis and wound closure [24]. A study demonstrated that H_2_O_2_ decreased the angiogenic ability of HUVECs through the VEGFR‐2 signalling cascade [25]. They revealed a decrease in the expression of angiogenic factors, including VEGFR‐2, HSP‐70, VCAM‐1, MMP‐9 and p‐Akt‐1, and a reduction in NO production, thereby inhibiting angiogenesis [25]. Additionally, superoxide anions–induced activation of the nuclear factor kappa B (NF‐κB) pathway can lead to the expression of antiangiogenic factors such as thrombospondin‐1 and endostatin, which antagonise the proangiogenic effects of VEGF and other growth factors [26]. Previous studies found that thrombospondin‐1 inhibits endothelial proliferation, migration and tube formation [27, 28]. Overall, the dysregulation of ROS levels in ischaemic tissues can disrupt the delicate balance between pro‐ and antiangiogenic signals, leading to the suppression of angiogenesis in limb ischaemia.

A strategy for treating limb ischaemia involves reducing ROS while promoting angiogenesis. Despite the widespread use of antioxidants to combat oxidative stress, free radical scavengers to eliminate metabolic waste and stem cells in cell‐based therapies, their clinical efficacy remains limited [29]. It is increasingly evident that HBO has been a beneficial adjunct in treating chronic diabetic ulcers of the lower limbs, while recent evidence on HBO for limb ischaemia remains ambiguous [10, 30, 31]. Understanding the molecular mechanism of HBO therapy is essential to proving the effectiveness of this technique. In this study, we elucidate the mechanisms that underlie the therapeutic effectiveness of HBO in enhancing angiogenesis following limb ischaemia. Our focus is specifically on its impact on ROS and the Nrf2/HO‐1/VEGF pathway. Firstly, our findings highlight the role of HBO therapy in ROS mitigation. ROS, generated as a consequence of ischaemic insult, contribute to endothelial dysfunction and impair angiogenesis. Although HBO therapy increases the quantity of dissolved oxygen carried by the blood, leading to a significant increase in oxygen concentration in body tissues, it also increases free radical production. However, it induces an antioxidant environment in circulation [32]. This antioxidative property of HBO therapy reduces oedema, decreases the levels of inflammatory cytokines, increases proliferation of fibroblast, increases production of collagen and promotes angiogenesis [33]. Based on the fact that inhibiting excessive production of ROS and reinforcing cellular antioxidant capability protect cells from oxidative stress injury, we hypothesise that HBO may protect HUVECs by upregulating antioxidases.

Secondly, our study delves into the molecular mechanisms of HBO therapy, specifically concentrating on the Nrf2/HO‐1/VEGF pathway. This pathway serves as a crucial cellular defence mechanism against oxidative stress by upregulating antioxidant enzymes and detoxification pathways. Nrf2, a basic leucine zipper transcription factor, regulates the production of antioxidants [34]. Under physiological conditions, Nrf2 is regulated by Keap1 bound to it. However, under oxidative stress conditions, Nrf2 dissociates from Keap1 and translocates into the nucleus, thereby mitigating the effects of oxidative stress by increasing the expression of endogenous antioxidant defence enzymes such as HO‐1 and NQO1 [34, 35]. A previous report has indicated that Nrf2 plays a crucial role in diabetic wound healing [36]. Another study has also documented the participation of Nrf2 during HBO therapy in traumatic brain injury [37]. Additionally, HBO treatment enhances endothelial tube formation by activating the expression of cytoprotective Nrf2 in human microvascular endothelial cells [38, 39]. Dhamodharan et al. found that HBO therapy promotes the healing of diabetic foot ulcers by increasing Nrf2 expression, thereby inducing antioxidant defence, promoting angiogenesis and reducing inflammation [22, 36]. In this connection, we attempted to investigate the effect of HBO therapy on Nrf2 and its downstream targets such as HO‐1 and VEGF in limb ischaemia. Our findings suggest that HBO intervention not only improves endothelial cells' ability to resist limb ischaemia–induced oxidative stress through the Keap1/Nrf2/HO‐1 pathway but also proves to increase the levels of angiogenic factors such as HIF‐1α and VEGF.

## 5 Conclusions

Hereby, we demonstrate that in mice following limb ischaemia HBO enhances angiogenesis associated with the mitigation of ROS accumulation. Additionally, HBO treatment enhances proliferation, migration and tube formation in HUVECs subjected to OGD injury. Associated with the upregulation of angiogenesis‐related proteins, including VEGFR, VEGF, Erk, Nrf2, Keap1, HO‐1 and HIF‐1α, HBO treatment rescues limb ischaemia‐induced endothelial dysfunction. Nevertheless, the translation of HBO to the clinical arena for promoting angiogenesis in patients with PAD requires further investigation.

## Author Contributions

**You‐Cheng Lin:** conceptualization (equal), investigation (supporting), validation (equal), visualization (equal). **Jhih‐Yuan Shih:** formal analysis (equal), funding acquisition (equal), supervision (equal), visualization (equal). **Yu‐Wen Lin:** data curation (equal), investigation (equal), methodology (equal), project administration (equal), visualization (equal), writing – original draft (equal). **Ko‐Chi Niu:** resources (equal), supervision (equal), validation (equal). **Chon‐Seng Hong:** resources (equal), supervision (equal), validation (equal), visualization (equal). **Zhih‐Cherng Chen:** software (equal), supervision (equal), validation (equal), visualization (equal). **Shin‐Chen Pan:** investigation (equal), resources (equal), validation (equal), visualization (equal). **Tzu‐Yen Chang:** resources (equal), validation (equal), visualization (equal). **Wei‐Chih Kan:** conceptualization (equal), investigation (equal), resources (equal), validation (equal), visualization (equal). **Wei‐Ting Chang:** conceptualization (lead), data curation (lead), formal analysis (equal), funding acquisition (equal), investigation (equal), methodology (equal), project administration (equal), resources (lead), software (equal), visualization (lead), writing – original draft (lead), writing – review and editing (lead).

## Conflicts of Interest

The authors declare no conflicts of interest.

## Supporting information


**Figure S1.** Effects of HBO on (A) body weight (B) food and water intake in mice with limb ischaemia injury. Data are presented as mean ± SEM. One‐way ANOVA followed by Tukey’s test was used to compare groups. *p < 0.05 compared with the indicated groups.

## Data Availability

The original data can be directed to the corresponding author upon reasonable requests.
