# Supplementary figures and images for: Hyperbaric Oxygen Therapy Improved Neovascularisation Following Limb Ischaemia—The Role of ROS Mitigation
Source: J Cell Mol Med. 2024 Dec 25;28(24):e70310. doi: 10.1111/jcmm.70310 (PMC11669187; doi:10.1111/jcmm.70310)

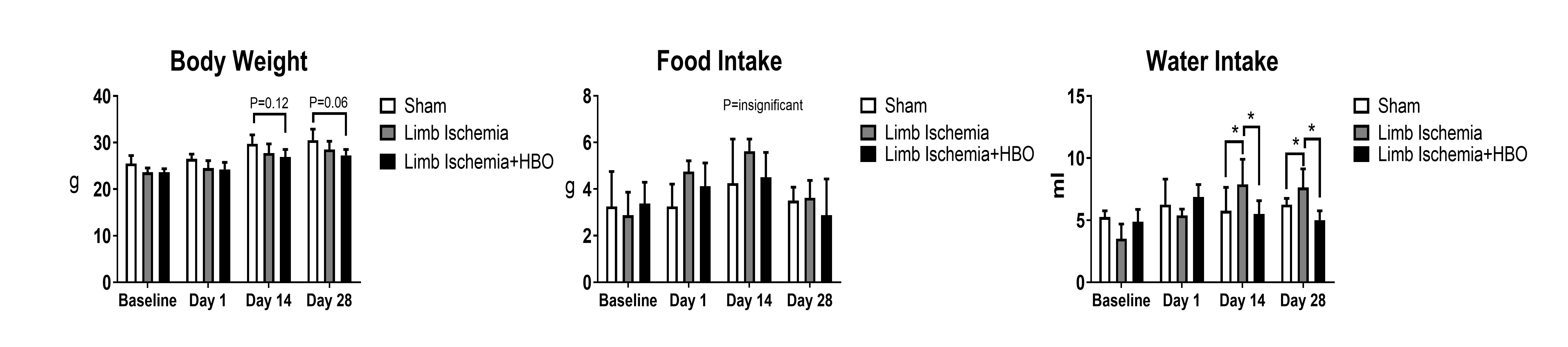

Supplement: Supplementary file 1 — Figure S1. Effects of HBO on (A) body weight (B) food and water intake in mice with limb ischaemia injury. Data are presented as mean ± SEM. One‐way ANOVA followed by Tukey’s test was used to compare groups. *p < 0.05 compared with the indicated groups. [file JCMM-28-e70310-s001.tif]
